# Case report: The remarkable response of pembrolizumab combined with RC48 in the third-line treatment of metastatic urothelial carcinoma

**DOI:** 10.3389/fimmu.2022.978266

**Published:** 2022-11-15

**Authors:** Zhenying Xu, Jiaman Ma, Ting Chen, Yu Yang

**Affiliations:** Department of Abdominal Oncology, West China Hospital, Sichuan University, Chengdu, China

**Keywords:** urothelial carcinoma, human epidermal growth factor receptor 2 (HER2),trastuzumab (herceptin), pembrolizumab, antibody-drug conjugates, RC48-ADC

## Abstract

**Background:**

Systemic chemotherapy has been the mainstay treatment for locally advanced or metastatic urothelial carcinoma (UC). In the past few years, novel immune checkpoint inhibitors (ICIs) and antibody-drug conjugates (ADCs) have improved the treatment of advanced UC.

**Case presentation:**

Here, we report systemic therapy of a 68-year-old male diagnosed with HER2 positive (immunohistochemistry 3+), programmed cell death ligand 1(PD-L1) negative metastatic UC, and renal insufficiency. He had encountered numerous metastases and failed first-line platinum-based chemotherapy and second-line treatment with pembrolizumab and trastuzumab. During third-line treatment with RC48 (a HER2 targeting ADC) combined with pembrolizumab, he achieved a rapid partial response (PR) in the first evaluation and subsequent complete response (CR) on PET/CT and long-term progression-free survival (>12 months) at the last follow-up on 25 August 2022. There are no grade 3 or 4 adverse events or aggravations of renal insufficiency during the third-line therapy.

**Discussion:**

RC48 combined with pembrolizumab demonstrated outstanding efficacy and safety in this HER2-positive metastatic UC patient. ADC combined with ICI is a promising anti-tumor strategy that deserves further exploration in advanced UC.

## Introduction

Urothelial carcinoma (UC) is a multi-origin malignancy originating from the urinary tract epithelium, including the renal pelvis, ureter, bladder, and urethra. UC is the most common cancer type of the genitourinary system worldwide. Platinum-based chemotherapy is the primary clinical management for advanced UC, which has shown slow development over the past 30 years. New treatment options, including immune checkpoint inhibitors (ICIs) and antibody-drug conjugates (ADCs), have recently significantly improved the treatment of advanced UC. Up to this point, the Food and Drug Administration (FDA) has approved pembrolizumab and atezolizumab to treat patients with locally advanced or metastatic UC. *Human Epidermal Growth Factor Receptor 2 (HER2)*, a typical oncogene in humans, is relatively highly expressed in UC. Disitamab Vedotin (RC48), an ADC designed for HER2, has demonstrated promising therapeutic efficacy in advanced UC and has received Breakthrough Therapy Designation from the FDA as second-line treatment for patients with advanced UC with high HER2 expression, including immunohistochemistry (IHC) 2+ or 3+. In January 2022, RC48 was endorsed by China’s National Medical Products Administration to treat locally advanced or metastatic UC patients with high HER2 expression (IHC 2+ or 3+) who have previously failed platinum-containing chemotherapy.

This article reported the excellent viability and well-being profile of pembrolizumab combined with RC48 as a third-line treatment for a patient with HER2 positive (IHC 3+) and programmed cell death ligand 1(PD-L1) negative metastatic UC and renal insufficiency who failed two cycles of second-line treatment with pembrolizumab plus trastuzumab.

## Case presentation

A 68-year-old male presented to the outpatient unit in December 2020 with upper abdominal pain. He was diagnosed with high-grade bladder UC 12 years ago. He underwent radical total cystectomy on February 5, 2010, and total urethrectomy for recurrence in the urethra on 25 April 2012. In April 2018, the patient sought care for a swollen left lower limb. The positron emission tomography and computed tomography (PET/CT) scan showed partial invasion of soft tissue into the left iliac vessels in the left pelvis. Subsequently, the biopsy pathology report confirmed UC metastasis. After receiving six cycles of gemcitabine plus cisplatin (GP) chemotherapy, he achieved a complete response (CR). However, he did not follow up regularly for more than 2 years from August 2018. In December 2020, the patient presented to our hospital for abdominal pain with enhanced CT showing foci in the right kidney and right ureter indicative of UC (4.6 cm × 6 cm) accompanied by multiple metastases in the liver (maximum of 6.6 cm × 4.4 cm) and lymph nodes in the abdomen and retroperitoneum. The liver biopsy results confirmed metastatic UC. IHC exhibited PD-L1 (−), MSH-2 (+), MSH-6 (+), MLH-1 (+), PMS2 (+), and Her2 (+++). The patient also had renal insufficiency with a creatinine of 109 umol/L. The renogram showed mild functional impairment in the left kidney and moderate-to-severe impairment in the right kidney. After receiving first-line treatment with gemcitabine plus carboplatin (GCb) chemotherapy on 25 December 2020, he achieved a partial response (PR) as measured using Response Evaluation Criteria in Solid Tumors (RECIST) Version 1.1. However, the tumor progressed after five cycles of chemotherapy because of enlarged hepatic metastases.

**Figure 1:**
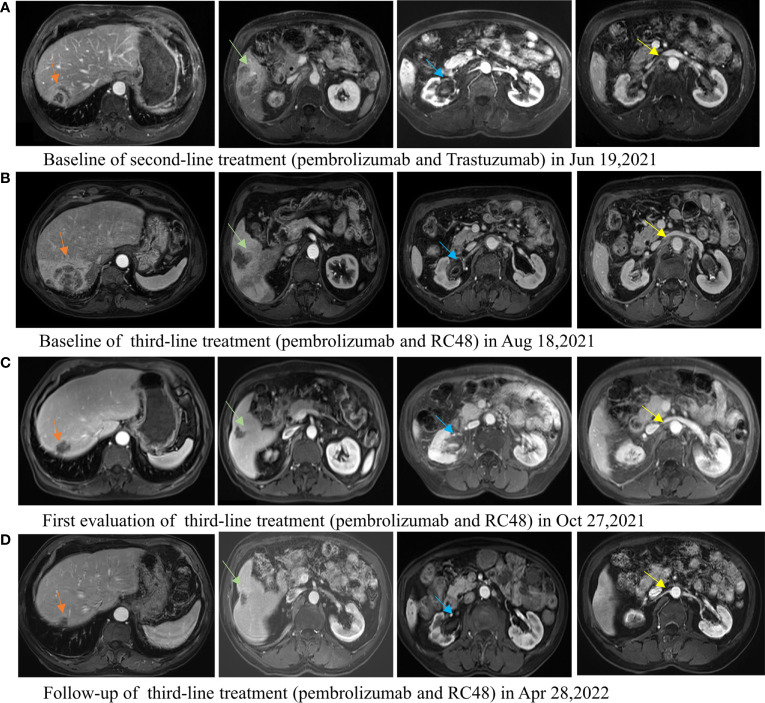
Lesions of the liver, ureteropelvic calyces, and lymph node change during the second-line treatment of pembrolizumab combined with trastuzumab and third-line treatment of pembrolizumab combined with RC48. **(A)** The baseline MRI image of the second-line treatment. **(B)** The image of tumor progression after second-line treatment, and the baseline image of the third-line treatment. The hepatic metastasis in the right posterior upper lobe was significantly enlarged and the other lesions reduced after second-line treatment. **(C)** The first efficacy evaluation after third-line treatment for 2 months: partial response. All tumor foci are reduced. **(D)** The image evaluation after third-line treatment for 8 months: partial response.

**Figure 2:**
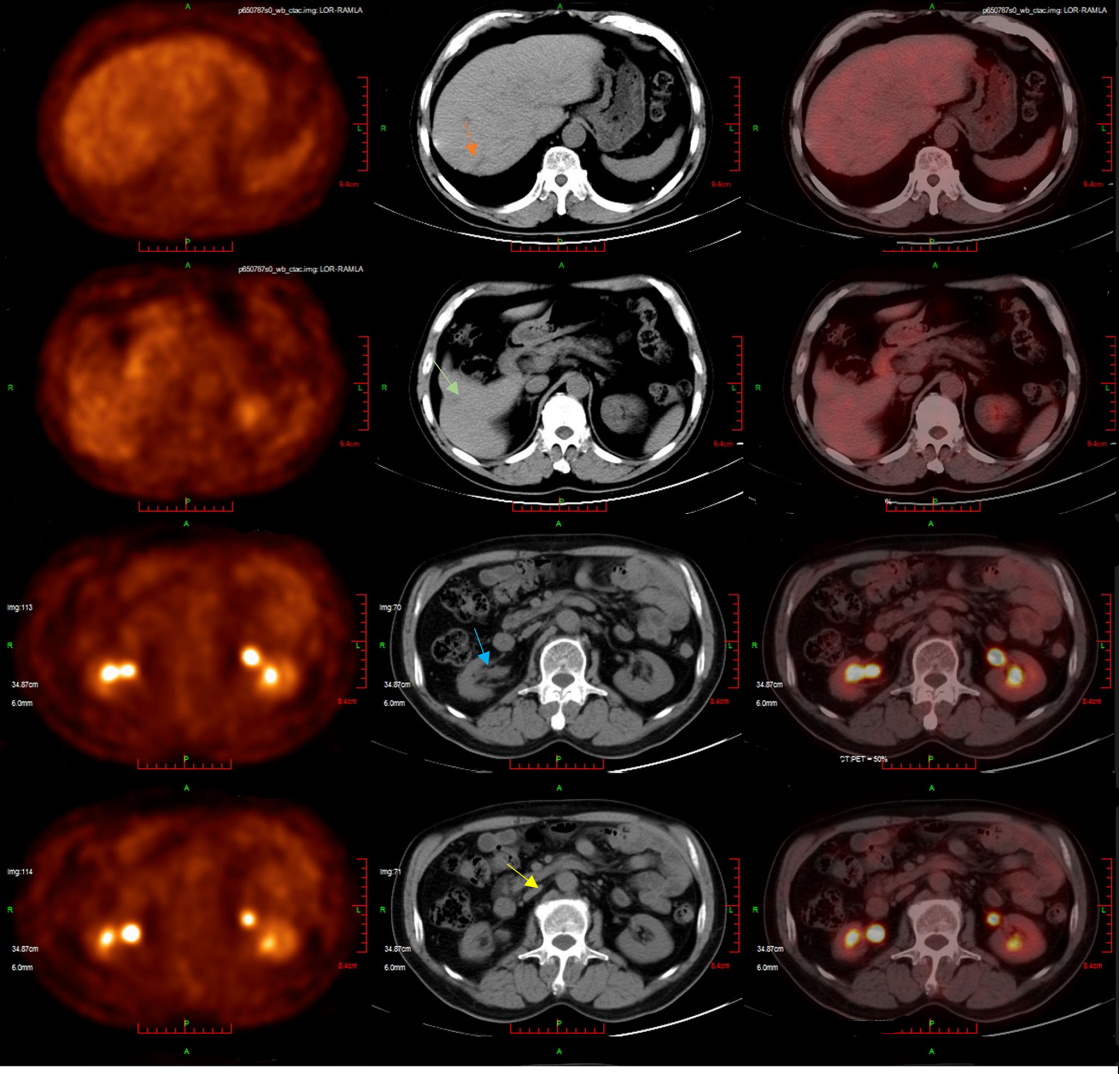
The patient’s latest PET/CT showed a complete response on 25 August 2022. Several slightly hypodense shadows were seen in the liver parenchyma, none of which showed abnormally high ^18^F-FDG uptake. No definite signs of residual tumors were seen throughout the body.

**Figure 3:**
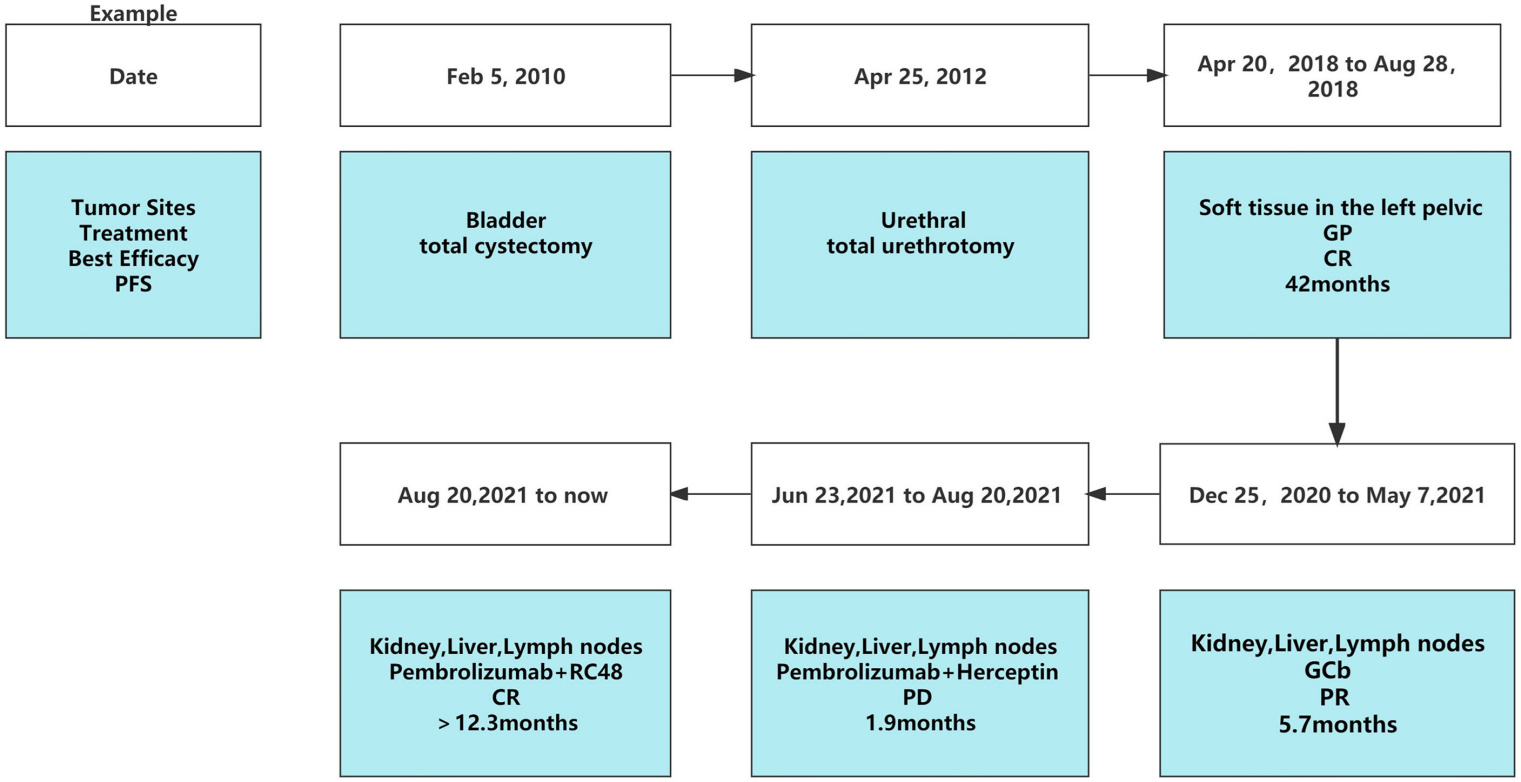
Timeline scheme of the major clinical events of the patient.

The second-line treatment involved trastuzumab 8 mg/kg loading dose followed by 6 mg/kg Q3W and pembrolizumab 200 mg Q3W from 23 June 2021 (**Figure 1A**). After two cycles, a magnetic resonance imaging (MRI) scan showed the tumor had progressed. On MRI, tumor foci exhibited heterogeneity in efficacy. Notably, the hepatic metastasis in the right posterior upper lobe significantly increased in size (6.6 cm × 5 cm), whereas the rest of the metastases of the liver and lymph node were reduced (**Figure 1B**). Third-line treatment with RC48 115 mg Q2W and pembrolizumab 200 mg Q3W started on 20 August 2021. After three cycles of treatment, the first evaluation achieved PR with a significant reduction in metastases in the liver (maximum size of 2.5 cm × 2.4 cm) and abdominal lymph nodes on 29 October 2021 (**Figure 1C**). The time to response (TTR) was 70 days. Due to the inconvenience of admission from another city, subsequent treatment continued about every four weeks. Imaging reexamined every 2–3 months showed sustained PR (**Figure 1D**). At the last follow-up on 25 August 2022, PET/CT was performed again. The report showed no definite signs of the tumor throughout the body, and hepatic lesions with low density showed complete remission of metabolism after anti-tumor treatment (**Figure 2**). The patient achieved CR. At this moment, the progression-free survival (PFS) in the third-line treatment was more than 12 months. No grade 3 or 4 adverse events (Common Terminology Criteria for Adverse Events 5.0) or aggravation of renal insufficiency was observed during treatment with pembrolizumab combined with RC48. In the initial stage of treatment, the patient experienced grade 2 vomiting with a weight loss of 10 kg, which may be attributed to RC48. Considering that the antiemetic effect of 5-hydroxytryptamine receptor antagonist was not obvious, he was given olanzapine 5 mg qd orally for a short time. The vomiting symptoms were quickly controlled and the weight gradually recovered. During the third-line treatment, the patient maintained a good quality of life. Currently, the patient’s performance status score was 0, and treatment is continuing. The main treatment steps for the patient are shown in **Figure 3**.

## Discussion

This case was metachronous UC with multiple origins. The patient had previously been diagnosed with UC of the bladder and underwent surgery in 2010, but HER2 status was unknown. After about ten years, he was diagnosed with right kidney and ureteral UC with liver, abdominal, and retroperitoneal lymph node metastases and HER2 positive (IHC 3+) through imaging and liver biopsy in December 2020.

HER2 is an essential therapeutic target of anticancer drugs. For HER2-positive (IHC 3+ or 2+ and FISH +) advanced breast cancer and gastric cancer, chemotherapy combined with trastuzumab is the indicated first-line standard therapy (1, 2). Studies have reported 6%–17% HER2 mutations and amplification in UC (3). However, in first-line and maintenance treatment, monoclonal antibodies (mAbs) and tyrosine kinase inhibitors (TKIs) for HER2-positive UC failed. A phase II trial (4) compared gemcitabine plus platinum chemotherapy with or without trastuzumab in advanced UC overexpressing HER2. No significant difference was found in median PFS (10.2 *vs*. 8.2 months, p = 0.689) or median overall survival (mOS) (15.7 *vs* 14.1 months, p = 0.684) between the two treatment options. In a phase III trial, lapatinib maintenance therapy showed no significant improvements in OS after first-line chemotherapy for HER1 or HER2-positive metastatic urothelial bladder cancer. In addition, the difference in PFS or OS was not associated with HER status (HER1-positive only, HER2-positive only, and IHC 3+ for HER1/2) (5). Therefore, the current systemic treatment options for advanced UC are not stratified by HER2 expression. For cisplatinum-tolerant patients, GP or methotrexate, vinblastine, doxorubicin, and cisplatin (MVAC) combined with granulocyte colony-stimulating factor (G-CSF) is the recommended first-line chemotherapy, with mPFS of 7.7–8.3 months and mOS of 14–15.2 months (6). Cisplatinum-intolerant patients can receive GCb chemotherapy (7) or immunotherapy with pembrolizumab or atezolizumab (8, 9). Pembrolizumab is the standard second-line treatment for advanced UC after failing platinum-based chemotherapy, with an mOS of 10.3 months and an mPFS of 2.1 months reported in the KEYNOTE-045 trial (10).

Despite the shortage of data about immunotherapy combined with trastuzumab on UC, our ideas mainly referred to the treatment progress of gastric cancer. In preclinical studies, trastuzumab has been shown to stimulate HER2-specific T-cell responses (11) and upregulate programmed cell death protein 1 (PD-1) and PD-L1 expression (12, 13). Coadministration of PD-1 inhibitors and trastuzumab enhances HER2-specific T-cell responses (11, 14). At the 2020 American Society of Clinical Oncology (ASCO) annual meeting, a Phase 1b/2 report combining pembrolizumab with trastuzumab and chemotherapy provided an ORR of 76.7% for advanced HER2-positive gastric cancer patients (15). Combining trastuzumab with pembrolizumab therapy was selected as the second-line treatment for our patient.

Unfortunately, in our patient with HER2-positive (IHC 3+) and PD-L1-negative UC, no synergistic effect was produced by combining trastuzumab with standard second-line pembrolizumab therapy. Interestingly, variable efficacy was observed between the enlarged right posterior superior hepatic metastasis and the reduced remaining hepatic and lymph node metastases (**Figure 1B**). Whether heterogeneity in HER2 expression existed among these metastases and affected the treatment response was unclear. Unfortunately, we could not verify this by biopsy of each metastasis. The short PFS of our case is also consistent with a later reported study (16). An analysis of 79 metastatic UC cases treated with second-line anti-PD-1 immunotherapy showed distinct differences in mPFS of 11, 3.7, and 1.8 months (p = 0.001) among HER2-negative (IHC 0), low-expression (IHC 2+7/FISH− and IHC 1+) and overexpression (IHC 3+ and IHC 2+/FISH+), respectively (16). HER2 expression levels increased from negative to overexpression with decreasing ORR (42.4% *vs*. 31.6% *vs*. 0%, p = 0.08). It was suggested that HER2 expression levels might influence second-line immunotherapy for UC. Also, in a phase 1b-2 trial, PANACEA (17) for trastuzumab-resistant, advanced, HER2-positive breast cancer, the synergistic efficacy of pembrolizumab plus trastuzumab was only observed in PD-L1-positive patients, with an ORR of 15% (6/40) and no remission in 12 PD-L1-negative patients. Therefore, the synergistic effect of pembrolizumab plus trastuzumab in HER2-positive cancers may differ depending on tumor type or expressed molecular marker.

RC48 (Disitamab vedotin) is a Chinese original HER2-targeting ADC, which has demonstrated excellent efficacy in advanced UC with HER2 IHC 2+/3+ in the phase II trials RC48-C005 (18) and RC48-C009 (19), reported in early 2021. For advanced UC patients who had failed at least one line of chemotherapy, the confirmed ORR was 46.9%–51.2%, and the mPFS was 4.3–6.9 months. The mOS was 13.9–14.8 months. In June 2021, RC48 was first approved in China for advanced HER2 overexpressing gastric cancer patients who have received at least two lines of systemic chemotherapy. In the phase II clinical study of advanced gastric cancer patients who were under at least second-line therapy, the ORR was 24.8%, the mPFS was 4.1 months, and the OS was 7.9 months (20). Subgroup analysis showed that the previous use of trastuzumab did not significantly affect the efficacy of RC48 for gastric cancer. Therefore, when our case progressed after trastuzumab combined with pembrolizumab in August 2021, RC48 was recommended. In the same period, the other two HER2-targeting ADC drugs, T-DM1 and T-DXd, had no relevant data on UC. The efficacy of apatinib for UC previously reported was not ideal. Besides that, considering the hepatic metastasis in the right posterior upper lobe was significantly enlarged and the other lesions reduced after treatment with trastuzumab combined with pembrolizumab, we also continued to use pembrolizumab in the third-line treatment.

Our case showed that RC48-ADC still works despite the failure of anti-HER2 monoclonal antibody therapy, which may be explained in several ways. Firstly, hertuzumab, a humanized anti-HER2 antibody, contained in RC48 is linked by a detachable linker to monomethyl auristatin E (MMAE) a microtubulin inhibitor. In addition to inhibiting the HER-2-receptor signal pathway, RC48 produces an anti-tumor effect *via* MMAE-induced cytotoxicity. Previous research also pointed to antibody-dependent cell-mediated cytotoxicity (ADCC) activity in *in vitro* assays (21–23). Secondly, compared with trastuzumab, hertuzumab has a higher affinity for HER2 (21). MMAE coupled with hertuzumab can potently kill HER-2 targeting tumor cells. Besides, MMAE released by enzymolysis has high membrane permeability, which can penetrate adjacent cells to produce a bystander effect and has a therapeutic effect on tumor cells with low or no expression of HER2 (24, 25). In the RC48-C011 study (26), for HER2-negative (IHC 0/1+) advanced UC patients treated with ≥1 prior systemic therapy, RC48 also showed promising efficacy, with an ORR of 26.3%. In the RC48-C008 study (20), the ORR of RC48 was 24.8% in trastuzumab-treated HER2-positive advanced gastric cancer patients who had received at least second-line therapy. Besides, RC48 and PD-1 inhibitors have a good synergistic effect, which is significantly higher than the efficacy of RC48 alone or PD-1 inhibitor alone (27).

As far as we know, this is the first report of RC48 combined with pembrolizumab in advanced UC. And more importantly, this case previously experienced failure of an anti-HER2 monoclonal antibody combined with pembrolizumab treatment. In our case, a phase Ib/II study (RC48-C014) reported a preliminary result showing promising synergistic efficacy of RC48 combined with toripalimab (an anti-PD-1 antibody) in advanced UC patients who were unable to tolerate or refused chemotherapy, or progressed after at least 1 prior systemic chemotherapy in 2021 (27). However, none of these patients received anti-HER2 monoclonal antibody treatment before enrollment. Seventeen patients enrolled (12 cases with HER2 IHC2+/3+ and five cases with HER2 IHC0/1+) achieved an ORR of 94.1%. At the 2022 ASCO-GU annual meeting, a phase 1b study of T-DXd combined with nivolumab also showed antitumor activity in UC patients with HER-2 IHC2+/3+ after the progression of chemotherapy, with an ORR of 36.7% (11/30) and mPFS of 6.9 months (28). For HER2-positive UC, HER-2-targeting ADCs combined with ICIs have shown good prospects, which deserve to be further investigated. Currently, a phase III study comparing RC48 in combination with toripalimab with chemotherapy in previously untreated HER2-expressing advanced UC is going on (NCT05302284).

## Conclusion

In summary, we reported that a metastatic UC patient with HER2 positive (IHC 3+) achieved a remarkable response, excellent PFS, and good tolerance when treated with pembrolizumab combined with RC48 after the failure of second-line treatment with pembrolizumab combined with trastuzumab. Although our report was only a case, it showed a significant difference in the efficacy of anti-HER2 drugs for UC. RC48 combined with immunotherapy has shown a very good prospect in advanced UC. It is worth further exploring in patients with low or negative HER2 expression and evaluating the efficacy of front-line therapy.

## Ethics statement

Written informed consent was obtained from the individual(s) for the publication of any potentially identifiable images or data included in this article.

## Author contributions

ZX, JM, and TC contributed to the material preparation, data collection and manuscript writing. Thesis was reviewed and revised by YY. All authors contributed to the article and approved the submitted version.

## Conflict of interest

The authors declare that the research was conducted in the absence of any commercial or financial relationships that could be construed as a potential conflict of interest.

## Publisher’s note

All claims expressed in this article are solely those of the authors and do not necessarily represent those of their affiliated organizations, or those of the publisher, the editors and the reviewers. Any product that may be evaluated in this article, or claim that may be made by its manufacturer, is not guaranteed or endorsed by the publisher.
